# The Past, the Present, and the Future: A Qualitative Study Exploring How Refugees' Experience of Time Influences Their Mental Health and Well-Being

**DOI:** 10.3389/fsoc.2020.00046

**Published:** 2020-08-21

**Authors:** Mette Sagbakken, Ida M. Bregård, Sverre Varvin

**Affiliations:** Faculty of Health Sciences, OsloMet–Oslo Metropolitan University, Oslo, Norway

**Keywords:** refuge, asylumseekers, mental health–related quality of life, mental health-state of emotional and social well-being, mental health, resilence

## Abstract

The experience of time has a decisive influence on refugees' well-being and suffering in all phases of their flight experiences. Basic safety is connected both developmentally and in present life with a feeling of continuity and predictability. Refugees often experience disruption of this basic sense of time in their home country due to war, persecution, and often severe traumatization, during flight, due to unpredictable and dangerous circumstances in the hands of smugglers, and after flight, due to unpredictable circumstances in asylum centers, e.g., extended waiting time and idleness. These context-dependent disruptions of normal experiences of time may lead to disturbances in mental life and extreme difficulties in organizing one's daily life. This article is based on narrative interviews with 78 asylum seekers and refugees in asylum centers in Norway, exploring their experiences before, during, and after flight. The distinction between abstract, chronological time, and concrete time connecting situational experiences (daily activities, such as daily rhythm of sleep and wakefulness) proved important for understanding how the experiences became mentally disturbing and how people tried to cope with this experience more or often less successfully. Prominent findings were loss of future directedness, a feeling of being imprisoned or trapped, disempowerment, passivity and development of a negative view of self, memory disturbances with difficulty of placing oneself in time and space, disruptions of relations, and a feeling of loss of developmental possibilities. Some had developed resilient strategies, such as imagining the flight as a holiday trip, to cope with the challenges, but most participants felt deeply disempowered and often disorientated. The analysis pointed clearly to a profound context dependent time-disrupting aspect of the refugee experience. An insecure and undefined present made participants unable to visualize their future and integrate the future in their experience of the present. This was connected with the inherent passivity and undefined waiting in the centers and camps, and with previous near encounters with annihilation and death. A response was often withdrawal into passivity.

## Introduction

“I am all that I inherited and all that I have acquired” (Mann, 1991).

Time is integrally bound up with our sense of identity, and different psychological definitions of personal identity tend to focus on the essential temporality of identity formation and identity development. This is, for example, described by Mann in his rather famous quote, which underlines the dynamics and continuity in identity formation. The subjective experience of time as a process is a basic human condition and connected with a feeling of being alive. This process gives a feeling of continuity and a feeling of some cohesion between the past, present, and future. This feeling of continuity is an experience both subjectively felt and socially and culturally determined by each group's relation to myths and rituals as well as socially organized linear time. The social organization of time gives the subjects the experience of developmental continuity connected with an experience of basic safety (Cipriani, 2013). Social phenomena and ways of organizing life specific to each culture creates a frame of reference that expresses the culture's time frame. The experience of time is thus socially determined (Sorokin and Merton, 1937), and ruptures in the social context (e.g., war, persecution) may threaten the individual's security. Developmentally, an individual's security is established early in reasonably safe attachment relationships (Schore, 1994; Crittenden and Landini, 2011), mediated through careful handling of the child's needs. Given adequate family and social contexts, the child will achieve a sense of safety that ensures the child that frustration and hunger will pass and thus install a feeling of hope for the future, a future-directed sense of time (Voort et al., 2014). A context-dependent basic sense of time is thus established from early development, with cultural and social differences.

When society and culture do not provide safe contexts, the family and, ultimately, the caregiver–child relation are put under stress. Wars, persecutions, and suppression create circumstances under which developmental possibilities, and thus a sense of predictability for children and adults in a society, deteriorate (Hollifield et al., 2018).

Forced displacement represents such a developmental risk. At present, there is the largest number of refugees since World War II, more than 70 million people (including internally displaced). The Balkan wars in the 1990s represented the beginning of an era with increasing numbers of people forced into flight. A significant proportion came to Western countries even if the majority were either fleeing in their own country (internally displaced) or fled to neighboring countries. The result was nevertheless a radical change in the policies toward refugees in the many parts of the Western world. For several reasons, the approach chosen by authorities was to frighten people from coming to Western countries and to hinder people in crossing borders (Gammeltoft-Hansen, 2016). This has resulted in increased aid to refugees near their home country, but much resources are now spent on border control and surveillance. Some refugees achieve the status of UN quota refugees and gain direct permission to stay, but the majority who try to reach Western countries has to rely on human smugglers. The consequence is that the flight has become increasingly dangerous with high mortality rates and high risks of traumatization (Silove et al., 2017; UNHCR, 2018).

People flee because they fear for their lives; often after their society has been severely damaged, relatives and friends killed, and life made impossible. Planning for the future becomes difficult, and basic feelings of safety and continuity of identity may be jeopardized. An experience of a possible future based on the experience of a reasonably safe past and a peaceful present that may give opportunities may seem impossible (Volkan, 2003). Thus, identity development and sense of time are put under pressure for large groups who are forced to flee or forcibly displaced.

Death is the ultimate end of time—and in itself unimaginable. When people die, they “pass out of earthly time” and in some cultures enter on a different journey where rebirth ensures a dimension of circularity of time (Gire, 2014). Under normal circumstances, the fact that people die is part of life, with appropriate explanations given, such as that it is “God's will,” “destiny,” or more technical medical explanations of the course of an illness. The fear of dying, of being annihilated at the hands of external malignant forces (terror state regimes, violent paramilitary groups, bombs from above, torturers, human smugglers, or drowning during flight) is qualitatively different from dying in times of peace, even if unforeseen. Such atrocities may evoke anxieties that may disturb identity development. We know that many torture victims live with a constant fear of annihilation, and that time will suddenly end (Lerner et al., 2016).

These fears and anxieties are connected with the wars and atrocities refugees have experienced in their home countries and during their flight through the deserts in Africa, boat voyages across the Mediterranean, imprisonment, slavery and sexual abuse along the borders of Western countries, and so forth (Grande, 2016; Silove et al., 2017). Such upheavals may be profoundly identity changing and cause a disturbance in the sense of time, often in the form of a shortened sense of a future (Beiser, 1987). Upon arrival in a potential host country, difficulties continue, especially in relation to the waiting time in asylum centers during the application process.

This article is part of a larger mixed method study seeking to identify resilience promoting and resilience inhibiting factors, on individual and contextual levels, among asylum seekers and refugees living in reception centers in Norway. Resilience is here defined as “*the capacity of a biopsychosocial system (can include an individual person, a family, or a community) to navigate the resources necessary to sustain positive functioning under stress, as well as the capacity of systems to negotiate for resources to be provided in ways that are experienced as meaningful*” (Ungar, 2019, p. 2). Resilience is thus a contextually dependent strategy to cope with hardship including temporal uncertainties (Ungar, 2008). During explorative qualitative interviews, the phenomenon of time was repeatedly addressed and strongly colored the interviews. It became clear that refugees' experience of time influences their mental health and well-being in various ways, and we decided that part of the study would explore refugees and asylum seekers' experiences during and after flight, with special focus on the role of time. It was also clear that there was a reciprocal influence between participants' mental health and the experience of time, but this was not focused on in this study.

## Perception and Experience of Time: When The Feeling of Time Breaks Down

To understand the role that the experience of time has in refugees' lives, we must look into some basic dimensions of time, especially the distinction between abstract, linear time, and concrete time related to daily, situational activities, which give an experience of regularity and predictability in daily life.

*Abstract, quantifiable time* as a basic dimension in our existence must be experienced and learned as a way of orientation. This time dimension provides conditions that enable us to intervene in the course of events by defining, realizing, densifying, and coordinating social activities (Johansen, 2001). Johansen (2001) underlines two salient characteristics of the modern form of time. First, time is homogeneous, implying that time is similar for all types of activities at all places. Thus, the present can be seen as a cross-sectional picture of a neutral time that encompasses the whole. Second, all modern time is continuous. It is not defined by actions or events, but is a dimension floating through and between activities in the form of periods or “rooms of time.” In other words, time is uniform and linear and transcends any situation. Yet, another characteristic is that humans, at least in the Western world, tend to think that time is available in a certain amount. Thus, our actions use parts of this amount, and we dispose, consume, exploit, or waste time. In other words, time is viewed as a resource to (and should) be used in different ways. In modern, capitalist society, time is therefore a force to be exploited rather than part of an action or event (Johansen, 2001).

On the other hand, *concrete time*, often called premodern time, is heterogeneous and discontinuous. This is time as connected with concrete, situational experiences, such as preparing food, organizing the house, making furniture, harvesting the fields, and so on. Time, in this connection, is not an abstract and independent dimension, and the abstract dimension of time somehow falls apart or cannot be taken into account as the action takes the time it takes. Thus, every experience, every set of actions have their own time. In other words, the homogeneous frame that time provides in a modern way of thinking is absent, which complicates the possibility of acting according to a common criterion. Time is built by concrete experiences, by commonly known cultural events, such as marriages, or by commonly known references, such as natural events, a sunset, or something that happened “5 winters ago.” This type of time cannot be separated from its content, since it is not anything but the events that it describes (Johansen, 2001).

These basic dimensions of time relate to the conceptions of synchronic and diachronic time. Synchronic time is the time that contains the flow of human action in a given situation, while diachronic time incorporates the movement in time (Nielsen, unpublished). Nielsen relates these dimensions to Johansen's conceptions of concrete (synchronic) and abstract (diachronic) time and points to the advantage of connecting concrete time to actions and events, since time is not just defined as a continuously moving point between past and future, but rather tied to the meaning of our actions. Thus, the present is an event or a social situation, not just a moment in a time arrow—a moment that in itself has temporality.

Nielsen also illustrates how, although abstract or diachronic time is linear, it is also heterogeneous, comprising a variety of “temporal strands, moving at different paces and in different spaces” (p. 5). Thus, diachronic time can be present in synchronic time and provide another depth and content. This idea of the multilayeredness of time and meaning is also found in psychoanalytical theories (Gutwinski-Jeggle, 1992). Thus, a person can be seen as layers of different interpretations, based on different points in time, existing as possible interpretations in a person's psychic universe (Nielsen, unpublished, p. 6). Memories of past experiences coexist with perceptions of present situations that are viewed in terms of representations of these earlier experiences, and earlier experiences may be reinterpreted in terms of new experiences (Varvin, 1997).

A person's subjective experience is, in other words, a temporal phenomenon, unfolding in the interaction between the past, the present, and the future (Nielsen, unpublished) in a specific cultural context. How these temporalities interact in a person's mind and how people interpret time are complex processes, personal, and idiosyncratic. In the interviews, however, we may observe the end result, the different ways our participants interpret both similar and dissimilar experiences of their flight destinies.

There are cultural differences in how time is conceptualized, which are reflected in social organizations of life, in myths, in language, and so forth (Boman, 1960; Ricoeur, 1984; Johansen, 2001). Most participants in our study come from non-Western cultures where, as in Semitic cultures, time is a part of life in the sense that what *has happened* tends to be an integral part of what *is*. In contrast, in Greek (and Western) thought, time is *part of life* that corrodes and finally destroys what is (Boman, 1960; Varvin, 2003). Even though cultural differences in how time is conceptualized among the participants are important, this has not been the focus in this study.

A person fleeing his/her home country needs, however, to adapt to situations with quite different and often strange (unknown) time frames, for example, situations where one has to act immediately to avoid danger, with no time to reflect; indeterminate waiting in queues, in camps in unknown places; and the experience of seemingly endless waiting for a decision on asylum applications. At the same time, the refugee needs to preserve a daily rhythm with sleep, eating, attending to bodily functions, and so forth. Such daily routines are related to home situations and family life and often difficult to maintain in life as a refugee (Varvin, 2016).

Thus, for refugees, the time dimension may be disturbed both on the abstract, chronological level, and on the concrete, action level.

*Abstract, homogeneous time* is disturbed during war and extreme upheaval as social institutions and coordination of social activities tend to be profoundly disrupted. This influences the organization of ordinary, daily life (concrete time) and also of maintaining a continuity of time, with a secure sense of connections between past, present, and future. *Concrete time* of action is made difficult or impossible in situations where daily activities may be dangerous or impossible. This becomes evident when homes are destroyed and people are forced to live in the streets or in shelters with constant dangers, or during flight, when refugees are dependent on others to organize daily life, often under dangerous and inhuman conditions.

## Aim

As time appeared in the material as a category of overriding importance, the aim of this study is to explore in what way the experience of time influenced refugees' mental health and well-being during and after flight.

### The Research Context

Asylum seekers apply for asylum upon arrival in Norway, and the waiting time varies from months to years, depending on the number of arrivals and country of origin. Reception centers in Norway are managed by non-government organizations (NGOs), private institutions, or the local municipality, and receive their commission and funding from the Norwegian Directorate of Immigration. Reception centers are located in all parts of the country. There are regular activities for the inhabitants, such as Norwegian courses, sports, handicrafts, etc., and kindergarten is available for a limited number of hours. However, activities vary from center to center, as do the quality and scope.

Asylum seekers are members of the National Insurance Scheme and are entitled to the same health services as nationals (Helsedirektoratet, 2017). Most centers have local nurses or interprofessional health teams, and primary health care functions (Rambøl, 2016; Helsedirektoratet, 2017). Formal and informal barriers to health care exist, however, especially regarding mental health care. These include inhabitants' lack of information on services, insufficient ability to express their mental health needs, health services' lack of ability to handle traumatized refugees, or rejection from the specialist health services on referrals (Varvin and Aasland, 2009).

## Methodology

This study presents qualitative data from the Norwegian part of a mixed method study with participants recruited in Norway and Serbia. Qualitative methodology was used to identify resilience-promoting and resilience-inhibiting factors on both individual and contextual levels, for asylum seekers during their stay at asylum reception centers in Norway.

A short, open, semistructured interview guide was developed. The guide consisted of three main questions, created to gather the participants' narratives describing difficult and helpful pre-flight, flight, and post-flight experiences, including their perceived and experienced quality of life from the time they decided to leave their home and until the interviews were conducted. Participants were asked to provide examples of situations, persons, or activities that have had an impact on them during their refugee journey.

Seventy-eight participants were recruited at five reception centers for families and single adults in different counties, rural and urban, and also located in regions far away from the capital in order to achieve maximum sample variation (Patton, 2002). An information meeting about the study, with translators, was held in all centers. Participants volunteered in connection with the information meeting or were recruited through center administration and staff or during informal conversations when the researchers spent time in the reception centers.

The inclusion criteria were refugees and asylum seekers living in asylum reception centers, above 18 years of age. Exclusion criteria were refugees and asylum seekers below 18 years or too ill to be interviewed.

A purposeful sampling strategy, aiming at maximum variation, was applied to obtain as many different perspectives as possible, i.e., variation in age, gender, family situation, education level, and ethnic affiliation. This ensured inclusion of the subgroups: families with children, single mothers with small children, single women, and refugees arriving as unaccompanied minors but tested to be adults (above 18 years). Some of the participants were below 18 (minors) when they arrived in Norway, but had become 18 while waiting for their application to be treated. Asylum seekers from Syria, Iraq, and Afghanistan were of particular interest due to their common flight experiences, but all asylum seekers were invited to participate.

The participants consisted of 28 women and 50 men, and the mean age was 29.9 years. Even though we sought maximum variation, we recruited more men, as there were often more men participating at the information meetings and men tended to be more accessible in the common rooms in the centers. Since women were less accessible, they were less available for informal conversations followed by recruitment.

Thirty were married and 34 lived with children in the center. Most had some education (primary school, 24; high school, 22; university, 26; and illiterate, 3). Most participants came from Syria and Afghanistan (38). The rest came from countries in the Middle East (Iran, 9; Iraq, 9; Turkey, 3; Palestine, 2; Egypt, 1; Kuwait, 1) and Africa (Eritrea, 4; Tunisia, 2; Ethiopia, 2; Congo, 2; Rwanda, 1; Egypt, 1; Zimbabwe, 1).

The participants all perceived themselves as refugees, and all had applied for asylum (47% application pending, 24% granted asylum, and 29% refused asylum). Length of stay in Norway varied (3 months to 10 years). A study under review, based on the quantitative part of the study, found that the mental health of the participants that were refused was significantly worse than that of the others (Grøtvedt et al., 2020), which is in accordance with other studies (Hocking et al., 2015). As we found that the time dimension had other and often more serious psychological consequences for those being refused asylum, we have chosen to address this particular groups in a separate paper.

### Data Production

The data production lasted from 2016 to 2017, altogether 18 months. Translators were used when necessary, by phone or with the translator present (English and Norwegian were used without translator). Interviews took place in a quiet room at the asylum reception centers and lasted 1–3 h. All interviews were tape recorded, with the participants' consent, and transcribed verbatim. Field notes gave information on setting, participants' reactions during the interview, and researchers' reflections following the interview.

The research included participant observation/field notes in the selected reception centers. In this way, we could move beyond selective perceptions and discover issues that were overlooked during interviews. This contextual knowledge facilitated a better understanding of what had been expressed during interviews (Fangen, 2010), such as conditions influencing resilience. Spending time with the residents by participating in daily activities at the centers contributed to building rapport and facilitated informal and formal conversations/interviews.

### Ethics

Informed written consent was sought from all participants. They were told that all information would be treated without name or any other direct identifiable information and that we would present the data in a way that secured confidentiality. They were informed that the research had no link to the asylum application process and that they could withdraw at any stage. Deidentification and confidentiality were ensured by using fictitious names, and other potential identifiers were also altered. Ethical approval for the study was obtained from the Regional Committee for Medical Research Ethics (REK) in Norway (REK 2016/65).

### The Research Team

The research team consisted of six researchers with different backgrounds: psychiatry, nursing, anthropology, and master students (within the field of nursing) under supervision. The different backgrounds allowed for negotiation from different perspectives in the process of interpreting the material, basing our analysis on researcher triangulation.

### Analysis

The chosen theoretical underpinning was phenomenology. This methodology attempts to understand the meaning of events and interactions within the framework of how individuals make sense of their world. As the interviews provided many and different expressions related to how time influences the participants' mental health and well-being, our intention was to explore the essential meaning of their descriptions. The data analysis was inspired by the principles of Giorgi's phenomenological analysis as modified by Malterud (Giorgi, 1985; Malterud, 2017).

We developed an analytical approach that relied on five interconnected stages: (a) familiarization; (b) indexing; (c) identification of a thematic framework; (d) interpretation: development of preliminary categories; (e) confrontation with existing theory on time experience by refugees; and (f) reinterpretation of themes, contextualization, and development of the final conceptual (or categories) framework.

The main researchers (authors of this article) also did interviews. In this way, the familiarization started during the interview process and was followed by in-depth reading of the interviews immediately after they were conducted. We all wrote down our first impression and reflections upon the interview, reflective notes that were available to all the authors. To ensure reliability, or consistency within the employed analytical procedures, identification of meaningful units and themes (indexing) was done through reading and rereading the interviews and notes in a collaborative process between the first and last author. Thus, this study is also inspired by hermeneutics, as we have obtained a constant and dynamic dialogue with the material, moving between perceiving and analyzing the material at a detailed level and by the authors trying to understand how the single pieces constituted broader themes. Through an interpretive process, a preliminary network of categories was developed, clustered around concepts based on the research questions for this study. The research questions were subsequently confronted with the material and refined. As it was important to see the gathered passages in context, rereading of whole interviews and transcripts from participant observations was done when considered necessary. Time appeared in the material as a category of overriding importance, something we had not anticipated. Subsequently, we identified, reviewed, and discussed a variety of empirical and theoretical sources addressing the phenomenon of time, as we found that the discourse on time has many ramifications. An interdisciplinary approach seemed fruitful, allowing for psychological as well as sociological and anthropological perspectives and theories in the process of interpretation (Cipriani, 2013). We gathered the passages relating to time in a table, giving us the main basis for the analysis. The following subcategories of the time category were developed:

loss of future directedness, entrapment, passivity and relation to self, memory, disempowerment, waiting for answer, disrupted time and friendship, loss of development, and strategies to cope with the time/interpretations of time. These categories will be elaborated below.

## Findings

In the material, refugees' encounter with the sudden and brutal possibility of “the end of time,” or a time experience as an unclear, unpredictable, and even feared dimension, seems to represent an analytical way of understanding the emergence of time as a recurrent theme. The extreme experience of losing a sense of future, characteristic for many inhabitants of asylum centers, must be understood partly on the background of the refugees' near encounter with the possibility of annihilation and death and also on the background of the inherent passivity and undefined waiting in the centers.

### Loss of Future Directedness

One of the most common issues expressed by the participants was how an insecure and undefined future left them unable to visualize their future life and also to integrate the future in the experience of the present.

Adem, a well-educated Syrian man in his late 20s, waiting for a response to his asylum application, used the metaphor of being frozen in time. He combined this metaphor with animal metaphors to illustrate inactivity and thus the inhuman character of his present situation:

Adem: Before, I was thinking about my future, right now I am not. Why do I have to think? I feel like I am a cow. Just eating and sleeping. I feel like a chicken, on the freezer. I am frozen. You know what they did—they (UDI) killed me, I am not kidding. UDI…UDI, they are not coming for me, like they say “okay, we will kill Adem” because they have something personal with me, no, but because they are really slow. Really slow…[…] I will just say to you, what would happen to you if you would stay without anything (to do) for ten months? What would happen? You can? You can't? I mean, like, maybe, but for me, I can't. “Cause for the first time, I stayed this really long time without doing anything.”

Like many other participants, Adem turned his frustration toward the rather abstract body of UDI (The Norwegian Directorate for Immigration) and claimed they had “killed him,” in the sense of processing his application too slowly and at the same time preventing him from doing meaningful activities. This passivity made Adem and many other participants turn their days into night, a sign of depression, according to Adem:

*Adem: How do you know if someone is really depressed? I go to sleep at five o'clock in the morning and I wake at four o'clock in the evening. Do you think that is a life? It's not! I never used to be like this. I'm always working. Studying. […] I don't want them to just give me a job tomorrow, no, but at least finish my papers (residency)…let me go to the school, at least. Let me do something*.

Helen, a woman from Eritrea in her mid-20s, also waiting for response on her asylum application, said the waiting time and the lack of activity made her feel mentally ill. She compared the situation at home with the present situation, and even though she faced severe challenges in her home country, she still felt her day-to-day activities in Eritrea were meaningful:

*Helen: I don't know how long I have to wait, and I cannot realize my plans (for the future). Because, just now it's like I am drifting in the air. I am doing nothing*.

INT: How does it influence you that you are drifting in the air?

*I feel ill. It influences my everyday life. Even if I had great challenges in my home country, I was still very active. I was doing activities, and I had two jobs, and besides that I did some teaching. […] It would have been nice if we were enabled to give something to the society, or go to school, rather than just sitting here*.

As described above, a recurrent theme was the need for doing something meaningful: activities, going to school, or working. The participants found being young and healthy, and still just sit passively and wait without contributing to society in any way, very difficult. Life tended to be described as “on hold,” and although some activities were offered in most reception centers, many experienced these as without meaning or direction. The resulting passivity was described as a large burden by many participants and may be linked to the near-death experiences of Adem, Helen, and many others. Thus, both Helen and Adam are in a system that, rather than alleviating fears, actualizes fears connected with loss of a future perspective.

### Entrapment

The prison metaphor was frequently used to describe the participants' feelings while waiting for a response to their application. Many compared this feeling to feelings they had in their home country or in camps in, e.g., Greece, of being trapped or imprisoned with none or very limited possibilities to act. Abdi, a Syrian man in his mid-30s, elaborated on how time spent in the asylum center compares to the feeling of being trapped he and other fellow asylum seekers experienced in the country they fled:

Abdi: The waiting is the worst for refugees. Some want to go to another country, being so tired of waiting. […] [Some are thinking about going back to their home country, or find a country where they can work…. We were trapped in Syria. We did not dare to go out due to the situation there. […] But then we managed to go to other countries […], but then we were trapped in an asylum centre. […] We ran from the war and the confinement there, but then we come to a new place where we feel confined… To have to wait for residency…to be able to come out in the society…. […]

I am so tired of this situation…the waiting. I will wait until April, then I have been here for a year, a whole year. […] And if I have not received residence permit then I have to do something else. I cannot wait here another year […] I am 35 years old, and the time is running…

Abdi seemed to say that, for him, being able to identify some point in time when he would be able to decide to pursue some kind of act helped him endure the present. Without really reflecting on the consequences, he seemed determined to do something soon with his own situation, something involving some kind of change to give him a feeling of being free.

Nizar, a Syrian man in his early 20s, was generally positive to the asylum seeker system and focused throughout the interview on the many fortunate and good aspects of his situation. However, when asked to describe the daily life of an asylum seeker, he pointed to the paradox of feeling imprisoned, although he was not:

Nizar: You (thinking)…You think that you are imprisoned, imprisoned in a very large prison. But you are not a prisoner, you are free. But you cannot do anything…

Thus, the feeling of being trapped or imprisoned was strongly tied to the feeling of being unable to change your own situation, implying a strong sense of loss of autonomy.

### Disempowerment and Unpredictability

A frequently recurring theme regarding time was the disempowerment inherent in having minimal influence on one's future, as well as one's present. The length of time spent prior to their potential recognition as refugees was experienced as a period that contained no predictability and allowed little or no influence in relation to decisions regarding the daily or future life. Firash, an Afghan man in his early 40s, provided the following perspective on his daily life:

*Firash: It is very tiresome (daily life). There is no specific plan for the days because we are not in a position to make plans; it is other people that are making the plans*.

INT: I see

Firash: Five months ago I applied for a table and a chair for me and my daughter…I have still not heard anything from the reception. […]

Firash continued to talk about his 10-years-old daughter, who he believes is depressed due to the situation. He said he had repeatedly asked for help but nothing had happened:

*Firash: My daughter has become depressed…She (a person in the reception centre) said that we should make an appointment and meet (to talk about the daughter), after that nothing happened. A couple of times she repeated that we were to have a meeting, but nothing happened. […] So everything has to wait…wait…wait…*.

Another dimension of the disempowerment experience was the total unpredictability of the waiting time. Participants said they would always get the same answer when calling to inquire about the progress; no one could say anything about time, not even whether it would involve weeks, months, or a year. Berat, a Turkish political refugee in his early 40s, who came to Norway with his family, said he had tried to phone several times without getting any answers. At the time of the interview, they had moved between four different asylum centers over a period of 6 months, their kids changing schools each time. At the time of the interview, they worried that the center they were staying in would close, forcing them to move again before the application was processed:

*Berat: We have applied […], and we have finished the second interview […], then now we are waiting*.

INT: Do you know anything about the time, how long…?

Berat: No actually. Even if you call them they say “everyone has their own process, so we cannot tell you the exact time,” but we don't know what is the level… (how long it potentially can take) […], we do not know anything about what is going on in our process…

A large number of participants emphasized that waiting without any known timeframe, knowing nothing about the application process, about any potential progress, were sources of extreme frustration.

### Passivity and Relation to Self

Some participants, both young and old, linked the lengthy wait to changes in the way they perceived themselves. Strong, emotional words and descriptions were used, like “hating myself,” “feeling hate toward myself,” “feeling unsuccessful,” “feeling like a loser.” These feelings were sometimes linked to suicidal thoughts. Adnan, an 18-years-old Syrian boy, who came as a minor but was moved to a center for adults, said he had waited 1 year and 2 months for the application to be processed. He described how he gradually started to view himself:

Adnan: You start to hate yourself by being here. You get tired of the clothes you are wearing…you get all these different thoughts…You want to kill yourself. You don't want to be here anymore. There is no school. I am new here. There is nothing to do…

Like many others, he continued to describe his present life using animal metaphors. Adan used first person “I” when he was active, but second person “you” when describing his passive position:

Adnan: I have told UDI to either give me a denial (the application) or give me a residence permit now. I have been here in one year and two months and that is a long time, and I could have done a lot during this time. And I have lived in many different reception centres, and living in reception centres is like living as sheep. Eating, drinking and sleeping, eating, drinking and sleeping…and then you get tired in the head of it. Sometimes it seems like my head is about to burst. Yes, quite simply explode…

As described by Adnan and others, living in a reception center may evoke a feeling of reduced worth in the sense that your life is comparable to the way animals are being kept. This comparison must be seen on the background of being exiled from one's country, implying a loss of protection and basic human rights. Many described extreme conditions in their homeland before fleeing, and many had experienced hardship and dangers during flight.

### Difficulties in Placing Oneself in Time and Space

Another topic related to time emerged in several participants' descriptions of confusion and lack of memory regarding both time and space. Awira, a Kurdish woman in her 30s, and her minor child had waited for their application to be treated for one and a half years. She struggled to place herself in time and space conveying her flight narrative. She described the long journey, spending time in different places, experiencing many hardships, saying this made her confused as to when and where events took place, her head being “very busy.” She could not remember when she left Kurdistan, but according to a date in her passport, it was about 3 years ago. She had problems describing where she had been at what point in time but elaborated on her time spent waiting in Greece, where she believed she had spent 1 year, but was not sure. She did, however, have a clear memory of the cold nights there, which reminded her of her present experiences. The similarities seemed to create a feeling of disintegration of time, and the continuation of the same experience was somehow transformed into a feeling of tiredness and self-hatred, similar to the feelings described by Adnan above:

Awira: It is a very, very heavy strain, and very, very huge strain, and heavy (the waiting). Because, when we came to Norway, we got the message “You are to be in the transit reception for one week, and after some three months you will get an answer.” But it became long…I think almost three months at the transit reception and… (pause) […] The situation is almost…impossible to endure…. Because, it is, I have experienced terrible things in Greece. It was cold, it was terrible situations. And then we came here […] I begin to hate myself, and I begin to be tired, and I don't think I will survive if it continues like this. I don't. […] I experience the same (as in Greece), I get headaches, I go out because I can't stand to be inside. I went to the doctor […], I need to talk to a psychologist… Here it is also cold. Cold and the darkness (polar nights)…I can't take it anymore…

The inability to visualize a complete narrative also related to many participants' descriptions of forgetting the past. While some described not thinking about the past as an intentional strategy of forgetting the past, many talked about memory loss in the sense of not being able to recall their home, past events, or skills and competencies related to their previous occupation. Damir, a young Syrian man in his early 20s, elaborated on this in an interview where we talked about his home in Syria:

I. Do you miss your home?

Damir: My home?

INT: Mhm. Do you long for it, miss it?

*Damir: I like my home, yes. I forgot my home*.

INT: You forgot?

*Damir: Yes, I also forgot Aleppo*.

INT: Have you forgotten it?

Damir: Yes, I forgot also Syria. I just remember Norway

[…]

INT: But do you dream about it […] do you remember difficult, bad things that have happened?

*Damir: No, no. I do not remember anything, no, nothing. I do not remember my house. My home, I do not remember. I forgot everything in Aleppo. I forgot Syria. Yes*.

INT: Do you remember the bombs and the war?

*Damir: No, no. Don't remember*.

INT: Maybe it is good that you do not remember?

*Damir: Yes, it's good. It's good for me*.

INT: But what about your family? Do they remember much?

*Damir: My family, I don't know. They don't remember either. My mother has forgotten. My mother forgot. My mother. Forgot. Also my sister forgot. My little brother, he remembers nothing*.

This may be interpreted as a description of a past too painful to remember at the same time as the experience of loss is painfully present. One may call this “known unknown,” typical for the many who have experienced unbearable losses, which they are incapable to contain mentally and express in a meaningful way to give a sense of something past. Thus, this kind of unbearable losses may become frozen and often wordless images in the mind. Past painful experiences are, however, represented in implicit/procedural memory as shown through bodily pains, confusion, and sensations connected with their present situation, e.g., the coldness, as procedural memory has no time dimension.

### Disrupted Time and Relations

Yet, another dimension of time, outlined by young people in particular, was that even the social part of the time spent in reception centers did not necessarily improve the experience of the waiting time. The majority of asylum seekers we met had moved between reception centers up to three to four times a year, making it difficult to establish social relations. The possibility of continuous friendship, meeting regularly and expecting that one will meet again soon, was lost. Thus, this time-preserving part of everyday life was disrupted by constant relocation.

Nizar, a Syrian man in his early 20s, described the situation for his younger siblings:

*Nizar: My brothers are […] 12, 15, and 16 years old. They are mentally tired of being in a reception centre. They don't want to talk with other people, because they are afraid that when they get to know them and get friends here, they have to move somewhere else. So, it is very difficult for children to be here*.

We also heard the stories of family members being sent to different parts of the country, although they begged to be allowed to stay together. One family chose to go to Norway because they already had close relatives living here. Upon arrival, they were told that they would be sent far north while their relatives, who already had residence permits, were living in the south. The family member participating in our study, Hassan, a well-educated man from Syria in his 40s, felt so frustrated and humiliated that he had decided to refuse any relocation:

Hassan: I will never go (up north). Because of how they treat us. I am educated, and so are the rest of my family. We can do anything (work) here. We have a priority to stay here (coming from Syria)…so why do they want to separate us?

He talked a lot about the response from the authorities to his decision not to move:

Hassan: Everyone is saying that “you will be out of the system then, you will be cut, your money will be cut.” But do you think I came here for the money? I did not! I am not here because of your money, I am not here because of your food. […] I am here because I am a human, and I want to be a human again… (referring to the war experiences)

Being “human again” related to many aspects of his situation, but Hassan related it first to the possibility of reuniting with his (extended) family, not having to move far away and thus lengthen the time until the family could be reunited. Being treated in such an inhumane way also prolonged his experience of not being human.

### Loss of Time and Development

Many participants described being unable to do anything, or to influence anything of importance, such as the application process, or work, as being similar to a “slow death.” Orhan, another Syrian man in his 30s, described the waiting process as particularly difficult due to the combination of doing nothing and the insecurity and fear that all that waiting would not be rewarded with a residence permit. He and others described how similar situations in the past, including waiting and insecurity (during flight), tended to melt in with the feelings in the present:

Orhan: To live in this uncertainty is killing, it is extremely painful… And not being of any use. Get up in the morning, drink coffee, and then you wait for a whole day, and then you go to bed… […] And the uncertainty… […] I get tired of not knowing […] I am afraid they will not approve my application… combined with the waiting… […] The waiting is killing because it reminds me a lot of the waiting in Greece. Even though it is not the same type of waiting, it is waiting for something uncertain. I get flashback from the past, the journey, from all the waiting…

The loss of time, through long waits and the postponement of future plans and dreams, was described as a source of anger and sorrow. Young people in particular often described time as a valuable asset to be protected, as something that could be “lost” and impossible to regain. Adnan, the 18-years-old Syrian boy who had waited for 14 months, expressed how time as a dimension in itself was perceived as valuable and how the loss of time was experienced as a loss of a part of life:

*Adnan: Before you come here (final destination) you think that life will change a lot. But when you come you become surprised that it is the opposite…that you continue to lose time. And that time is valuable, it is a part of our life*.

Many refugees, from Syria in particular, categorized themselves as “legal refugees.” They said they had expected to be processed more quickly due to their well-known refugee status and were disappointed by the extensive time used to clarify their status. Jamal, a Syrian man in his late 20s, said he gradually lost his feelings of motivation and happiness during the wait:

Jamal: When we got to know that we were to go to Norway, we became very happy. And I started to rest a bit…watching different things about Norway on YouTube. And I started to learn some Norwegian on my own. […] But then we came to Norway, and everything is so slow here. The whole system in Europe is slow. So, our lives are put on hold and I have lost my courage. It's like I am not motivated to learn Norwegian now; I have become so disappointed over the situation…

Some said they felt treated like “criminals” when the application process took so long even though all their papers “were in order” and the situation in their home country “well-known.” Having to put life on hold this way was described as a provocation and a humiliation; to Adem, this justified his unwillingness to participate in activities at the reception center:

*Adem: I'm not going. Never, to any activity. […] Others, they are going fishing, they are going to the mountains, they are going to, I don't know where, I'm not. I'm not in the mood*.

INT: Because you just feel like you're lacking energy, or…?

Adem: Yes, yes… […] I'm not really…. I feel like I'm just wasting my time, I'm just like, they are cheating on me, okay? “We will keep him silent to just go to these activities and he will be happy.” I feel like that…it doesn't mean that it's true, but I feel it's like that…

Adem said he would not give in. He felt participation meant giving up his personal freedom, giving in to those he felt cheated him. As he was treated like someone undeserving of refugee status, as a potential criminal, he might as well behave like one by not complying with the expectations of the system. His reaction did, however, seem to represent a deeper form of resistance; he could not comply with a system that he perceived as suppressive and which he felt was destroying his possibility to be a person. His strategy may be seen as counteracting the experience of being dehumanized.

### Strategies to Cope With Temporal Uncertainties

There were several ways of dealing with the dimension of time and related feelings of insecurity, waste, passivity, sorrow, humiliation, and anger. Some emphasized preventing meaninglessness by maintaining a daily rhythm; others pointed to activities that facilitated group solidarity. Some underlined the need to make sense of the present, while others told how thinking of the (better) future was the only way to endure the present.

Adnan, the 18-years-old Syrian boy who came as a minor and had developed a negative view of himself and his life, told how he tried to handle all the difficult thoughts that had accumulated. Focusing on the future, thinking that everything might change tomorrow, he somehow managed to cope:

Adnan: It is not like I am tired of my life all the time, I give myself a chance. I think about tomorrow; “I will get a residence permit tomorrow,” and then the next day is coming and then I say “ok, I must give it another chance.” Then (the next day) I will get a residence permit…

While Adnan focused on tomorrow, Damir, the young Syrian man unable to remember Syria, also waiting for an answer on his application, exemplified another strategy. He emphasized the difference between him and his friends in trying to make sense of daily life by engaging in activities that connected him to the present as well as the future:

INT: What do you do here during daytime (at a centre near the coast and surrounding mountains), do you exercise or something?

*Damir: No, just bicycling. And walking in the mountain*.

INT: You go to the mountain. Hiking?

Damir: …and I go to see the ocean. To see the ocean also…

[…]

INT: Do you fish as well?

*Damir: Yes of course, every day*.

INT: You fish every day?

Damir: Yes

*INT: Wow, that sounds nice*.

*Damir: I will go today, after coming back from school […]. Coming back, and then having dinner, and then go fishing*.

INT: OK. Together with friends or?

*Damir: No. Alone. My friends are sleeping (looking serious). They sleep until 0300 PM or 0400 PM*.

INT: Why?

Damir: Because they don't sleep at night

INT: Why do they not sleep at night?

*Damir: Because they don't have school, they don't go to school, and then …it's boring. Yes. They don't go to school. Only I go to school*.

INT: Why don't they go to school?

*Damir: Because, as my friend said: “I don't learn anything. Learn nothing. I just go to classroom and I'm there.” And the teacher is talking, the teachers speaks […] He don't understand anything*.

INT: OK. Because he can't speak the language?

Damir: No, he can't. No. And he remembers nothing (the language)…

Damir continued to talk about the importance of sleep; of creating a rhythm enabling him to be active during daytime; this enabled him to pursue activities that gave meaning to his everyday life, investing in the future by going to school and learning the language. He told how he worried about the passivity he saw not only among his friends but also within his own family, him being the only one who was active during daytime and the only one who had learned the language (the interview was done in Norwegian).

Karam, a Syrian man in his early 20s, developed a similar strategy. He had just received his residence permit and was engaged in different activities, such as a local sports club. He told how he had been thorough with his schoolwork, investing in his future, a future that recently had become more clarified. He had, however, long before he got his residence permit, engaged in activities:

INT: Can you say something about how it has been in the different reception centres?

*Karam: Yes, it has been good, I have been good, a good situation there. And I do understand that this is temporary, to live in an asylum centre*.

INT: Are there any other activities you do during the day?

Karam: Yes, every day I go to the sea. For two years, I have been going to the sea, yes. For two years. […]

INT: To fish, or…?

Karam: Yes

[…]

Are you alone often? (he has mentioned being lonely earlier)

Karam: Yes

INT: Mhm. How is it?

*Karam: It is…I am used to it. It is perfectly normal*.

INT: Mhm. Is it boring?

Karam: Yes, so I just use the whole day doing different things. Yes. Different activities, like going to school, and then do some reading, then go out hiking, then go to the sports club, that sort of things…

As illustrated by Karam, even with significant variances between the reception centers in type and amount of activities being offered, some participants always seemed to find things to do, even when alone or different from the group they were somehow associated with.

Like Karam, many emphasized the need to acknowledge the temporality of the situation, and ambitions and hope of a better future helped preserve a positive focus while waiting.

Yaser, a Syrian man in his early 30s, waiting for his application to be responded to, underlined the importance of enduring and accepting the situation, even if you suffer at the present:

*Yaser: When you have identified a goal you also have to endure a lot, and you cannot complain too much. You know it is a bad situation, but it is temporary*.

Some developed strategies of reinterpreting time, aiming to experience the present as meaningful or less insecure and threatening. Carlito, a well-educated man from Syria in his late 20s, provided this perspective:

*Carlito: I remember that I stayed at one place, at the border between Greece and Macedonia, and there was rain. It did not stop for many days […] and we woke up all the time, the tent being full of water, and we had to leave the tent and try to get rid of the water. […] The conditions were very difficult, very difficult. It was something that I had not experienced before, but mentally I still felt very good there, actually, and the things we contributed with was very good. […] We organized many different activities, and we took responsibility for the cleaning for example. […] We identified those speaking good English and they taught refugees English. And, then some would teach Kurdish for example. Because the Kurds live in four different countries and are not allowed to go to schools that teach in Kurdish, and then we thought that it was good that we were teaching Kurdish. […] Personally, every day I interpreted for people going to the doctor and followed two to three persons every day to the doctor*.

*[…] There were also refugees that could play an instrument, that could sing, and then they would teach the others in singing, in dance and in music and playing instruments. And all these activities were for free. The reason why we did this was that we were told that the border was closed. So instead of just waiting for the border to reopen, we thought that life must go on. We cannot just sit here and wait for the borders to reopen, because that may take time. It can take one year, it can take two years, and if one start to think like this, you will have mental difficulties, and that is why we thought that we had to do something. We had to fill our time. We had a lot of time. We have to use our time in a sensible way, and that is what we did*.

Carlito described trying to make people think differently about the situation, not only as a coping strategy but also as a way of viewing the present time and the present opportunities:

*Carlito: Ok, let's think that this is a tourist trip. We are on an adventure […], we have to learn something, we cannot sit here and wait for the borders to reopen […]. I went to a Greek school for four months and I learned to speak a bit Greek. And learning something new, it does something with a human being; that you have not “used up” (misused) your time, but that you have learned something. That is a very good feeling. And then I came here (Norway), and I think about all the good days there, even though there was little food, little money, suffering, and bad conditions… Sometimes I slept on an empty stomach…. But mentally I felt fine…*.

Cemil, a Turkish political refugee in his late 30s, described a similar strategy; he and his wife changed the interpretation of time and space by pretending to be in some sort of holiday camp. This way they were able to fill the present with meaning and joy while gradually revealing the part of reality that included the future for their children:

*Cemil: We were playing a game with our children actually. Even while we are asking for asylum they were thinking it is a bank office or something. […] As a family, we encourage each other. We try to make each other strong […] so maybe it was a good thing we said we were camping (laughing)*.

INT: And did they believe that?

*Cemil: Yes, they believed it is a kind of camping. After a time, they see the reality…. They speak with each other, what is happening, they ask questions. We try to explain them step-by-step*.

Keeping focus on the here and now, in a temporary (holiday) camp setting, seemed to enable a focus on the positive aspects of the present, avoiding a continuous negative focus on an insecure and unpredictable future.

Carlito continued to elaborate on how he tried to encourage people to reinterpret their focus while spending time waiting for the application to be processed:

*Carlito: I came to this reception, and here there are many depressed asylum seekers and refugees. Many of them have waited long for a residence permit. […] I tried to encourage them by saying that residence permit is just a paper. It is nothing more than a paper. What will happen if you do not get a residence permit? Are life going to end? You have to live your life, you cannot just sit here and become depressed and wait for the permit*.

Carlito's own coping mechanism was to focus on life, outside linear time, and realizing the future might not bring what he expected.

## Discussion

### Perceptions of the Past, the Present, and the Future

The interviews showed time as an important dimension in how the participants viewed and interpreted their present situation, crucial for their well-being and mental health. The participants frequently expressed how an insecure and undefined present made them unable to visualize their future and to integrate the future in their experience of the present. The unpredictability in the post-migration phase clearly disturbed their ability to tie the past and the present to the future (Haberfeld et al., 2019). This must be understood not only on the background of the inherent passivity and undefined waiting in the centers but also on background of refugees' previous near encounters with annihilation and death (Kovras and Robins, 2016; UNHCR, 2018). Such dangers evoke deep and overwhelming anxieties, which may or may not develop into a post-traumatic condition but will be stored in memory and may emerge later. Such anxieties force the person to focus on the present to remain attentive to possible dangers, causing emotional dysregulation, frequently seen in post-traumatic conditions (Nickerson et al., 2015a).

As a rule, such situations of imminent danger are radically different from earlier experiences. Our participants may not easily recognize these dangers as part of something known. Boat trips were, for example, described as utterly new and terrifying experiences, for which they had not developed coping strategies. Such situations were somehow similar to waiting in receptions centers, with little or often no available mental guidance for evaluating the present situation. The focus on the present tended to dominate, detached from earlier experiences. Without guidance, it was difficult to know what may happen next, disturbing the sense of a reasonably secure future. This loss of anchoring in time and space, expressed by many of our participants, may signal mental disintegration in people on the verge of mental breakdown (Rosenbaum, 2000; Varvin, 2003). Many described the future as somehow non-existing or unclear, making them unable to visualize a complete narrative of the past, present, and future (Varvin, 2003), like the Kurdish woman unable to remember any timeline for her flight or the Syrian boy insisting he had “forgotten” his home.

Being unable to tie the past to the present, and the present to the future, seemed to create a sense of uncertainty and meaninglessness, which may cause impairment in the sense of reality; cognitive organization of perceptual impulses becomes extremely demanding as it becomes difficult to distinguish important from unimportant (Puvimanasinghe et al., 2014). The material shows that being repeatedly trapped in similar situations—enduring long waits in a refugee camp, then in an asylum enter—being as cold in the host country as in the refugee camp—seems to bring the past into the present, and the present into the past in a way that melts and reinforces previous experiences (Palic et al., 2015). In other words, these multilayered experiences, connected to different time points in a linear time, melt together in a way that disturbs the possibility to scatter the flow of actions both on the abstract, chronological level and on the concrete, action level.

### Prisoners of an Unknown Time

A response to the confusing intrusions of past difficult experiences was often withdrawal into passivity, giving an impression of hopelessness. We saw how this condition was reinforced by inactivity and waiting, connected both with the flight experience and with life as an asylum seeker. Studies among asylum seekers show that high levels of depression and trauma are linked to the indefinite and temporary nature of the asylum process (Mansouri and Cauchi, 2007), and research shows, not only among refugees, that experiencing time as passing slowly is linked to mental suffering (Flaherty et al., 2005; Biehl, 2015; Horst and Grabska, 2015).

Some participants described life in a reception center as inducing a feeling of reduced worth, comparing their lives to that of animals. This must be seen on the background of being exiled from one's country, losing autonomy, protection, and basic human rights. Studies and reports show that refugees are regularly treated in inhuman ways and that such dehumanizing experiences may set their imprint on feelings of self-worth (Nickerson et al., 2015b; Grande, 2016; Varvin, 2017; Kingsley, 2018). Based on this, the use of animal metaphors to describe the passivity and hopelessness in asylum centers becomes more comprehensible, a continuing feeling of dehumanization, without citizenship, no autonomy or control after arrival in the host country, may reinforce the negative feelings with regard to self-worth.

The prison metaphor was also commonly used when describing time spent waiting in different reception centers. This metaphor, like the animal metaphor, can be understood as illustrating a feeling of loss of autonomy and subsequent disempowerment, reduced possibilities to plan, to act, and make decisions regarding the future. The metaphor also illustrates a feeling of unjustifiably being “criminalized.”

Studies on prison inmates show that prisoners experience time as a source of suffering in itself (Medlicott, 1999; Wilson, 2004), and prisoners' perceptions of the present, past, and the future show that the present tends to be extended and the past and the future tend to be distorted (Medlicott, 1999). As pointed out by Griffiths et al. (2013), asylum seekers differ from prisoners in that their future destiny, including when and if they are granted asylum, and in which context they are going to live, is highly uncertain. Additionally, the legal framework and related practice may suddenly change—the immigration system in Norway and other countries being in flux. In other words, in contrast to prisoners, familiar with both space and time, asylum seekers lack the privilege of having a “sentence” with time and space defined (Griffiths et al., 2013). Not having any idea of how long or under which conditions they are going to be detained significantly shapes the way they experience the waiting. An overwhelming majority in our material described the waiting time as painful, sometimes incomprehensible due to the well-known situation in their home country (e.g., Syria) and sometimes totally unbearable. These findings are consistent with other studies among refugees. For example, in a mixed method study from Attica, Epirus, and Samos in Greece, the epidemiological survey showed that between 73 and 100% of the refugees suffered from anxiety disorder. The qualitative part of the study showed how the refugees overwhelmingly reported experiencing uncertainty and lack of control over their current life and future, causing psychosocial distress and suffering (Bjertrup et al., 2018).

Similarly, in an ethnographic study on mental health implications connected to the process of resettlement among Iraqi refugees in Cairo, the refugees spoke of exile as “living in transit”—a condition that led to an altered experience of time in which the future became particularly uncertain, and where life in general was perceived as highly unstable (El-Shaarawi, 2015).

Research on temporary protection mechanisms among refugees shows that immigration policies can restrict people by creating temporary migration stages (Gammeltoft-Hansen, 2016). All stages include waiting for decisions, be it when and to which asylum center one will move next, interviews with the authorities, needed technical aid, or an appointment with a doctor. The temporal uncertainty is reinforced when waiting for decisions related to the present or near future is added to the waiting for major decisions related to the future, such as whether the application is granted, when decisions are to be enacted, when identity documents are to be sent, if one are to have a future in the present country, and if so, where and under what circumstances (Griffiths et al., 2013). These latter time stages, however, are often so long and unpredictable that they may be characterized as permanent temporariness (Bailey et al., 2002; Simmelink, 2011). As outlined by Griffiths, a significant source of powerlessness and insecurity for the asylum center residents is the constant tension between anticipating time ruptures through constant changes (as having to move to a new asylum center) and fearing indefinite and long periods of inactivity, uncertainty, and feeling unsafe. Both “suspended time” (lack of change/waiting) and “temporal rupture” (dramatically shifts) can separately and combined create chaos (Griffiths, 2014).

Daily routines and activities give a person a sense of continuity and safety, while disruptions of routines, as in situations of sensory deprivation, conversely create loss of contact with reality, a tendency toward autistic thinking and loss of continuity (Harrison and Newirth, 1990). There is thus ample evidence for the importance for mental health of being able to maintain the “normality” of daily routines. Thus, when many refugees are trapped or “imprisoned” in a situation with some sort of suspension of time as the world around them continues forward (Griffiths et al., 2013), this may cause serious disturbances on aspects of mental functioning. In this trapped situation, the priority for many will be maintaining a daily rhythm, giving an experience of synchronic time. This will, however, prove extremely difficult as some sense of linear time with a feeling of a past and a future contextualizing the present are necessary for the ability to uphold synchronically anchored time activities (Johansen, 2001).

### Responses to Temporal Uncertainty

Our study shows the variety of ways in which the participants responded to their situation. Gasparini's (1995) distinctions between three different types of waiting may be useful in shedding light on these different responses (Gasparini, 1995). He describes how people may view waiting more or less purely as an obstacle to action; waiting may constitute an experience filled with substitute meaning, and waiting may be perceived as a meaningful experience. Waiting perceived as an obstacle to action, as a loss of time and a difficulty in maintaining a feeling of synchronic/cyclical time, as if life itself stopped, was by far the most common expression among the participants in our study. This loss was expressed through feelings of powerlessness, insecurity and fear, humiliation, as well as provocation. Lack of meaningful activities and enforced idleness seemed to reinforce these difficult feelings; many participants expressed what can be described as desperation in regard to not being allowed to do something, paid or unpaid, while waiting.

Being unable to relate to the future, and consciously or unconsciously suppressing the past, many seemed to enter into some sort of empty synchronic (cyclic) time—time was repetitively empty, with no future-oriented activities. The social aspects of daily life also become difficult when moving frequently, causing unpredictable interruptions to social relations. Several participants described an intentional strategy of avoiding attachment or socializing with others at the reception centers, being afraid that such relations might soon end. Research has shown that inhabitants in asylum centers initially form family-like groups with brothers, sisters, and even some parental figures. The breaking up of these family groups is regularly experienced as a serious loss, which again may evoke experiences of earlier losses (Goosen et al., 2014). In the study from Attica, Epirus, and Samos in Greece, it was found that disruption of key social networks in addition to an absence of interactions with the surrounding Greek society led to psychosocial suffering. Feeling isolated combined with the general passivity of life in the refugee camps aggravated feelings of meaninglessness and powerlessness (Bjertrup et al., 2018). Thus, instead of social relations representing a meaningful and time-preserving part of everyday life, they may instead represent a threat of loss, discontinuity, disruption, and feelings of not belonging.

Being hindered in living with a normal linear time perspective, establishing social relations and some sort of production in accordance with time, may cause not only a feeling of discontinuity and being “in transit” but also represent transformation into an abnormal liminal state. Such a liminal phase may influence a person's identity; the identity may partly be lost or disrupted and may remain unclear for an indefinite time (Ball and Moselle, 2016). An ethnographic study from Texas, USA, exploring Syrian refugees' narratives of forced displacement and resettlement, found that refugees continued to suffer while waiting for permanent resettlement. Many waited for years, as the application process was long and complex, and entered into a period of liminality. The concept is used to describe how the identity and well-being of a person suffer from having no ties to a place for an unpredictable period of time, and how the ambiguous state of the person causes feelings of loss and confusion regarding their identity markers (Mzayek, 2019).

If we move back to Gasparine's three different ways of handling waiting, we see examples from the material that some participants somehow managed to accept being in a liminal phase, partly by acknowledging the temporality of the situation and partly because they adjusted by identifying activities that kept the present from being empty. Some were also, irrespective of their present status regarding the application, conscious of focusing on activities related to the future, such as learning the local language and prioritizing schoolwork. Thus, the waiting was a situation filled with substitute meaning, in combination with meaning that partly integrated a future perspective. This way of handling waiting was also illustrated in the study of Syrian refuges in Texas, where the long resettlement process gave several opportunities to develop what the author call “resilient tactics.” Many men would, for example, help sustain their families by continuously trying to engage in income-generating activities, while women supported their children by teaching them at home when they could not attend school. Some actively sought to establish friendship with local community members trying to reconstruct their identities by incorporating identity markers from their temporary host countries (Mzayek, 2019).

Yet another approach, illustrated in the findings by some participants, was taken by those who described the waiting as a meaningful experience. A few participants told how they managed to find meaning being immersed in the present, focusing on filling the present time with action, some even consciously separating themselves from thoughts oriented toward the past and the future. To understand this, and why it seemed to provide comfort and meaning, we can look at George H. Mead's (1863–1931) understanding of the relationship between action and time. He underlines that the present time can only provide meaning if it consists of temporal differences (past and future) and that human action can represent a tool that can achieve this temporal difference. Action is not to be understood as a movement in time *per se*, but rather as a means to manage time, develop time, and experience time. Any action produces time in the sense that it develops temporal differences between the present, the past, and the future; all actions, even though appearing in the present, immediately become an event and a reference point for oneself and for others. Such an event creates temporal differences, such as before/after, cause/effect, objective/agent, past/future, and the like, which encourages responses, reflections, and thus some kind of development of a personal narrative. Mead's main point is that without managing the present time, by creating these temporal differences, one may become pacified and paralyzed in the sense that one does not manage to connect events in daily life in a way that provides direction or meaning. This is somehow similar to the point made by Nielsen (unpublished), who underlines the importance of connecting concrete time to actions and events. Time, in this context, does not consist of points on a time arrow but of situations that have its own temporality. Thus, time becomes not only a dimension that continuously moves between the past and the future but also tied to the meaning of the action the person is conducting. In other words, the present is an event or a social situation that can be used to create temporality. We may say that some of the participants that seemed to manage well also seemed to be able to redefine time, or their understanding of (the value of) time, by using the present time actively. In this way, they seemed more capable to tie concrete and abstract time, thus reducing some of the pain involved when synchronic time was interpreted as “lost.”

Norbert Elias (1887–1990) points to time as a tool to ensure a steady point for orientation in modern society. Time helps provide logic and meaningful contexts, for example in situations of suffering and lack of meaning in the present, converting these into an expectation of future happiness and meaning. In other words, time becomes a means through which we can achieve our goals; thus, time is also an important component in a (continuous) production of a personal narrative/identity. Elias sees time as “mentally civilizing” by its ability to structure and discipline modern human beings' orientation and self-regulation (in the Freudian sense). Thus, feeling like one is “drifting in the air,” without managing to see or establish temporalities in present situations and to identify achievements or goals within a certain timeline, could help understanding the mental state of many of the participants. This state was sometimes characterized by strong and emotional descriptions like “hating myself,” “feeling hate toward myself,” “feeling like a looser,” and sometimes also linked with suicidal thoughts; this is in contrast to those who managed to find meaning and happiness and feel “mentally fine” by orienting themselves toward activities in the present.

As there are few studies on refugees' experience of time in relation to mental health/suffering, insights from studies of prisoners may throw light on refugee experiences. A qualitative study from the UK (Medlicott, 1999), exploring suicidal prisoners in the light of the pains of prison time, found that suicidal prisoners experience time as an acute source of suffering and strongly associated with the deterioration of their sense of self. Participants referred to the “pains of empty time” including the expectation of more of the same pain in the time to follow and “without any chronology of events” to mark or distinguish the time to come. The author underlines that the difficulties in managing time has no necessary relationship with the length of sentence nor the time spent in prison; this is because the ability to handle time varies significantly between individuals.

Medlicott refers to suicide statistics from the 1990s in which 40% of those that took their own lives in prison were on remand, meaning that they had not yet been convicted and that they may be acquitted or receive a non-custodial sentence. Those who managed to cope with the prison time, including those serving life sentences, were those who did not let time become an overruling dimension in their life, but who managed to exercise autonomy over their own time. They achieved this by accepting the timeframe as well as actively acquiring knowledge of what the frames, shaped by the time and space, allowed in terms of self-development. This strategy is similar to the participants in our study that seemed to cope better than the others. Instead of allowing all influence and autonomy to be taken away, these individuals found creative and autonomy-preserving ways of still influencing the experience of their time and their situation, thus showing a capacity for future directness and a way of dealing with temporal uncertainties. Even though patterns of strategies were identified among our participants, it was difficult to see any relation to age, gender, or other markers explaining why some managed better than others. In a meta-ethnography of sources of resilience in young refugees, including 26 empirical studies, it was found that even though refugee groups from different countries show many similarities in their sources of resilience, the resilience processes have several individual and culturally based components. For example, for some of the young refugees, access to school provided both hope and social support, and they were positively challenged to succeed their education despite adverse challenges, while others became discouraged as adversity increased over time; these findings that are in line with our study (Sleijpen et al., 2016).

### Structural Power Relations

Another lens for understanding the different interpretations and strategies unfolded in this material is viewing time as a tool in structural power relations (Foucault, 1975). Foucault's description of “states of emergencies,” including strict physical regulations and loss of temporal autonomy in certain circumstances (e.g., outbreaks of contagious diseases, political riots), has similarities to what can be interpreted as space as well as time discipline in asylum centers: structural forces seeking to discipline people that represent a threat to order and normality in a society within the frames of segregated institutions that limit people's possibilities to maintain their autonomy, to act and to invest in personal development, all exercised within a timeframe that one does not have any influence over and within a bewildering immigration system constantly *in flux* (Griffiths et al., 2013). Historically, such disciplinary measures have seldom been directed toward the privileged members of society, but rather imposed on groups without power, such as poor people, ethnic minorities, and refugees (Foucault, 1975; Lupton, 1995).

An example of the suppressive and disciplinary nature of the asylum system is addressed in the autobiographical account of Behrouz Boochani's, No Friend But the Mountain. Being a Kurdish journalist, Boochani sought asylum in Australia but was instead illegally imprisoned for 5 years in the country's most notorious detention center on Manus Island. He describes guards and nurses wearing uniforms as reminiscent of dystopian regimes, and how the fenced and isolated detention center serves as disciplinary measures, “pacifying even the most violent person…” Similarly, to a prison, the detention center maintains its power over time as well as the power to keep people in line. In his analysis, he draws attention to interconnected social systems of domination and oppression, illuminating how subjugation and degradation of refugees is facilitated by a more comprehensive idea of Australia's colonial imaginary and the prevailing xenophobia in the society (Boochani, 2018).

In a study of refugee accommodation in Athens, Berlin, and Copenhagen, the term “Campization” was used to capture the structural forces embedded in the design of the accommodations (Kreichlauf, 2018). The description highlights that refugee migration is deeply related to discourses on crime, terror, and a general view that refugees represent a threat. Refugee accommodations are sites through which these logics materialize, and the secluded and segregated organization produces limited possibilities for life. As the length of stay is mostly unknown, the camps are described as existing between the temporary and the permanent (Kreichlauf, 2018).

Thus, time as a perceived tool of power can be used to understand the degradation and humiliation felt by many of the participants having to comply with the rules of the system, including long waits to have their application treated, lack of information about the progress, a context of forced idleness, having to move from place to place at short notice, often involuntary separated from extended family members while waiting. Furthermore, like prisons, the time discipline and regulation of bodies are combined with a ruthless emptying of time, the time being slow and relentless, representing a potent tool for what can be perceived as a measure of discipline or punishment by a powerful agent (Medlicott, 1999). This same authority has the power to fill or not fill the time with meaningful activities, to speed the process of providing answers, and to help family members remain together.

As we have seen in this and other empirical studies, the possibilities for counteracting powerlessness and mobilize resilience in the sense of “*navigate the resources necessary to sustain positive functioning under stress”* (Ungar, 2019, p. 2) are present, even during the most suppressive circumstances. Boochani in Manus Island demonstrated “resilient tactics” by refusing the role of being a supplicant in a suppressive system. Instead, he uses art to secure a connection with the future, as art will have the chance of living beyond its contemporary moment. He secures his identity as a journalist by reporting about the conditions on Manus Island on smuggled mobile phones. Similarly, as found in our study, resilience is expressed through people resisting the system by opposing decisions of movement in time and space, by creating alternative or imagined realities, and by people actively searching for meaningful relations and activities related to the present and the future.

## Concluding Remarks

Migration control authorities and related control policies can play a direct role in sustaining or creating temporal uncertainties and ruptures in the present as well as in the future. As argued by Griffiths et al. (2013), this power is masked as well as exacerbated by time-consuming bureaucratic procedures, which tend to conduct strict categorization and evaluation of a person's immigrant status, while at the same time encouraging the picture of the “ideal immigrant” who patiently, submissively, and *disciplined* comply with a sequence of events from arrival to settlement. However, as noticed among the asylum seekers in our material, some wait for years to receive a final answer while others get their answer quickly, again in the context of a bewildering system in which one has no influence and in which one's future destiny lies in the hands of others. In other words, the troubled and emphasized relation to time must partly be understood as a continuation and reinforcement of unsafe and dehumanizing preflight and flight experiences. It must partly be understood as the result of an insecure and undefined present situation, making people lose the ability to visualize their future life, thus integrating the future in the experience of the present: Furthermore, it must partly be understood on the basis of timeframes being totally unpredictable and beyond individual control, implemented in a context that may be seen as a system with excessive use of disciplinary measures.

Further research is needed, however, to find what conditions and circumstances that can make more personal control and resilient developments possible under the restricted conditions asylum seekers and refugees live under.

## Data Availability Statement

The datasets generated for this study are available on request to the corresponding author.

## Ethics Statement

The studies involving human participants were reviewed and approved by Regional Committees for Medical and Health Research Ethics (REC). REC South East-Secretariat: 2016/651 Mental health and quality of life among asylum seekers and refugees. The participants provided their written informed consent to participate in this study. Written informed consent was obtained from the individual(s) for the publication of any potentially identifiable images or data included in this article.

## Author Contributions

MS and SV were responsible for the conception of the study and development of the study design. MS and SV was responsible for drafting the manuscript, while all authors have contributed with relevant perspectives by critically revising it. All authors participated in the data collection, and the data analysis. All authors read and approved the final manuscript, and are accountable for all aspects of the work in ensuring that questions related to the accuracy or integrity of any part of the work are appropriately investigated and resolved.

## Conflict of Interest

The authors declare that the research was conducted in the absence of any commercial or financial relationships that could be construed as a potential conflict of interest.
